# Testing the validity and reliability of the Matching Familiar Figures Test-2021: An updated behavioral measure of reflection–impulsivity

**DOI:** 10.3389/fpsyg.2022.977808

**Published:** 2022-11-10

**Authors:** Ralph E. Viator, Yi-Jing Wu, Allison S. Viator

**Affiliations:** ^1^Rawls College of Business, Texas Tech University, Lubbock, TX, United States; ^2^Dallas Art Therapy, Dallas, TX, United States

**Keywords:** Matching Familiar Figures Test, information sampling task, cognitive reflection test, reflection-impulsivity, heuristics-and-biases, cue processing, actively open-minded thinking, need for cognition

## Abstract

The Matching Familiar Figures Test (MFFT) is a well-known and extensively used behavioral measure of reflection-impulsivity. However, the instrument has several deficiencies, including images designed for school-age children in the United States during the 1960s. Most importantly, an adult version of the instrument is currently unavailable and the lack of a single repository for the images raises questions regarding the MFFT’s validity and reliability. We developed a 21st century version of the MFFT using images that are familiar to adults and reside in a freely accessible repository. We conducted two studies examining validity and reliability issues. In Study 1, participants interacting with the MFFT-2021, versus those interacting with the original MFFT20, spent more time on the task, took more time in making their first response, and were more likely to complete the task without errors, even though the average number of errors was higher than the comparison group. The coherence of these results is evidence of convergent validity. Regarding predictive validity, the MFFT-2021 remained a reliable predictor of rational thinking, such that participants who demonstrated more reflection (less impulsivity) tended to avoid rational thinking errors. Also, performance on the MFFT-2021 predicted higher quality judgments in processing job characteristic cues with embedded interactions, a form of configural information processing. We also found evidence of concurrent validity: performance on the MFFT-2021 differed in a predictable manner for participants grouped by their performance on the Cognitive Reflection Test. In Study 2, we tested discriminant validity by comparing participant performance on the MFFT-2021 to their performance on the Information Sampling Task (IST), another behavioral measure of reflection-impulsivity used in studies of psychopharmacological and addiction behaviors. For our participants (undergraduate business students), we found that the MFFT was a stronger predictor of performance on rational thinking tasks, and, contrary to prior studies, our exploratory factor analysis identified separate factors for the MFFT-2021 and the IST, supporting discriminant validity, indicating that these two instruments measure different subtypes of reflection-impulsivity.

## Introduction

We examine the validity and reliability of an updated version of the Matching Familiar Figures Test (MFFT-2021). The American Psychological Association defines the Matching Familiar Figures Test as follows:

A visual test in which the participant is asked to identify from among a group of six similar figures the one that matches a given sample. Items are scored for response time to first selection, number of correct first-choice selections, and number of errors. The test is used to measure conceptual tempo, that is, the relative speed with which an individual makes decisions on complex tasks (American Psychological Association, 2022).

The MFFT measures cognitive style along a dimension that varies from reflective (a preference for accuracy over response speed) to impulsive (a preference for responding quickly and less concern for accuracy; Stanovich, 2009; Baron et al., 2015). The initial 12 figures for the MFFT were developed by Jerome Kagan and colleagues for a series of studies in the 1960s that focused on information processing by elementary school age children (Kagan et al., 1964). Examples of the figures can be found via Internet searches using the key words “matching familiar figures images.” Over time, concerns emerged regarding whether measurements from the original MFFT were reliable and suitable for older children. Cairns and Cammock (1978) addressed those concerns by developing a longer and more reliable measure, a 20-item MFFT, which avoided floor and ceiling effects previously documented (Salkind and Nelson, 1980; Cairns and Cammock, 1984).^1^

Since the 1980s, the 20-item MFFT has been utilized in numerous studies that measure the tendency for reflection vs. impulsivity in people of all ages, including adults, as well as children. For example, using the MFFT, researchers have examined the relationship of reflection-impulsivity to attention deficit hyperactivity disorder (Young, 2005; Bramham et al., 2012; McDonagh et al., 2019), pharmacology issues (van Wel et al., 2012; Fikke et al., 2013), aggression behavior (Sanchez-Martin et al., 2011), intellectual disability (Rose et al., 2009), metacognitive functioning in young adolescents (Palladino et al., 1997), risk-taking behavior (Perales et al., 2009; Young et al., 2013; Herman and Duka, 2019), use of illegal substances (Morgan et al., 2006; Huddy et al., 2017), pathological gambling (Toplak et al., 2007), computerized adaptive testing of university students (Wang and Lu, 2018), brain lesions (Berlin et al., 2004), and avoidance of rational thinking errors (Viator et al., 2020).

In spite of extensive use of the 20-item MFFT for 40+ years, several concerns are apparent. First, as clearly stated by Quiroga et al. (2011, p. 86), “There is no alternate form for adults.” Whether images designed for children, are reliable in triggering and measuring reflection in adults is a concern. Participants might respond impulsively if they are bored and become disengaged, thus responding quickly and incorrectly. Second, the figures developed by Kagan et al. (1964) and Cairns and Cammock (1978) contain outdated images, such as telephones with mechanical dials, women’s dresses with unfamiliar styles, architecture from the 1950s, and unrecognizable eyeglasses. Such outdated images might not trigger reflection in 21st century adults. Third, a central repository for MFFT images has not been available for several decades. Researchers often cite that they are using the MFFT20 developed by Cairns and Cammock (1978) without reference to a handbook or website that provides access to the 20 sets of figures. Thus, academic studies might be using different figures and inconsistently measuring reflection-impulsivity, raising concerns regarding the MFFT’s reliability. To address these three concerns, we pursued generating a 21st century set of images that met three criteria: (1) the images must be oriented for adults and adolescents (2) the images should fit into the culture of the 21st century, and (3) the images must reside in an easily accessible repository that includes computer code for interfacing with widely used psychological software.^2^

**Figure 1:**
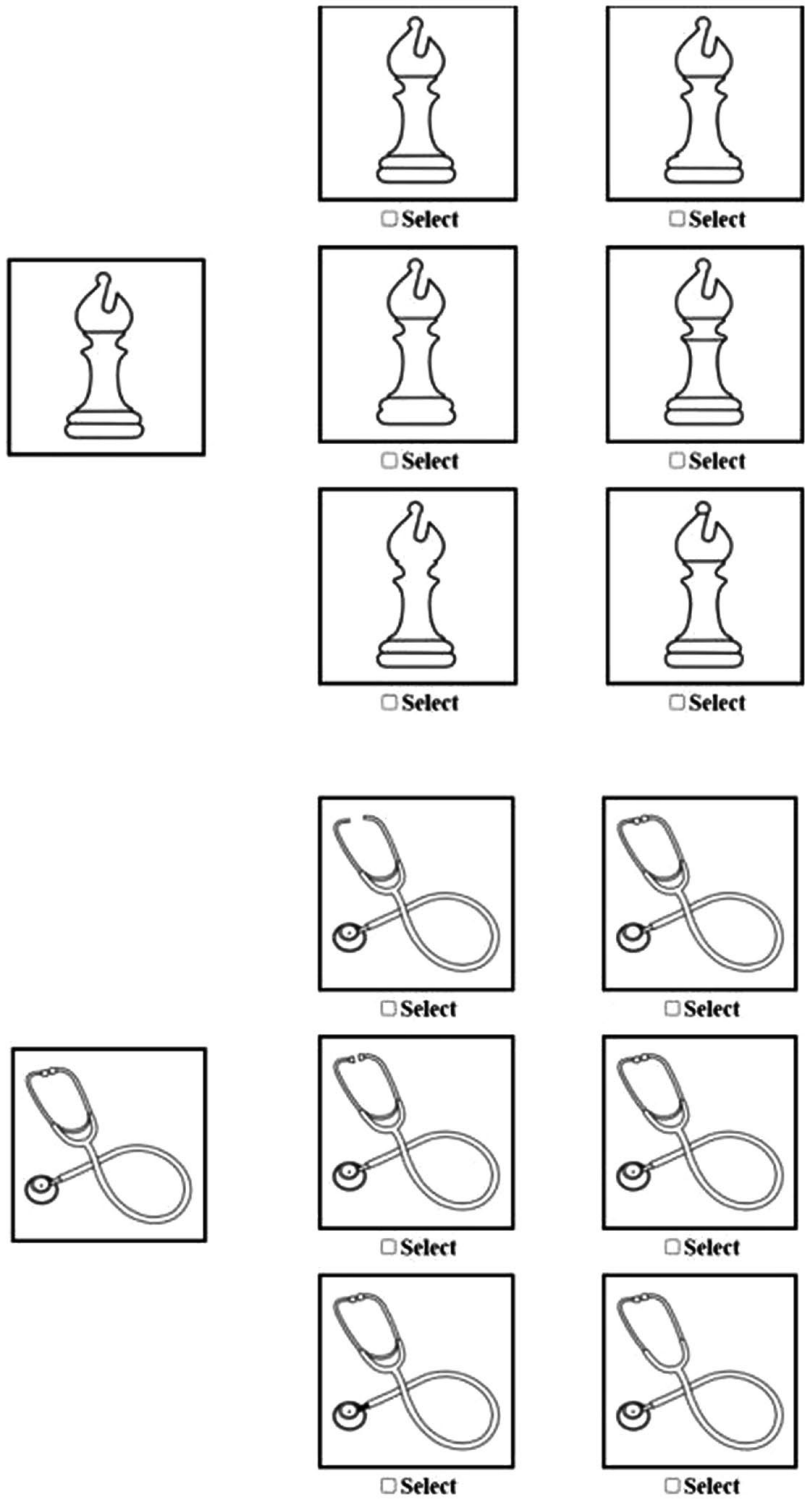
Example images from Matching Familiar Figures Test (MFFT)-2021.

We refer to the updated figures as the MFFT-2021, identifying the year generated and initially tested. Examples of two sets of figures are provided in Figure 1. In developing the new figures, to the best of our ability, we retained features and “rules” used to distinguish one figure from another in the original MFFT, such as figures with missing pieces, inverted shapes, disproportional shapes, changes in shading, as well as additional lines and missing lines.^3^ The new figures, and previously utilized figures, are available for viewing at the following website.^4^^,^^5^ Further, using the tools available at www.PsyToolkit.org (Stoet, 2010, 2017), the authors have created a repository of the images and computer code, which are available at Open Science Framework.^6^ The zip file can be downloaded to a personal computer, uploaded to a user’s PsyToolkit account, and then run as an experiment on PsyToolkit.org, generating a spreadsheet reporting MFFT performance for each participant. We conducted two studies examining validity and reliability issues, as outlined in Trochim et al. (2015).

- Study 1 tests convergent, predictive, and concurrent validity issues.
- Study 2 tests the discriminant validity of the MFFT-2021 by comparing its performance to the Information Sampling Task. Study 2 also serves as a test of reliability.

## Study 1

In order to assess the validity of the MFFT-2021, we obtained comparison data from the study by Viator et al. (2020), who used a 20-item version of the MFFT based on images found *via* Internet searches and references from previous research (Kagan et al., 1964; Cairns and Cammock, 1978; Carretero-Dios et al., 2008; Caswell et al., 2015; Ramaekers et al., 2016). This comparison data establish a baseline for measuring whether the MFFT-2021 enhances participant engagement, or not. Also, we examine whether the MFFT-2021 is a reliable predictor of performance on two different tasks: avoiding rational thinking errors (using the heuristics-and-biases composite reported in the Viator et al., 2020), and, evaluating potential employer job characteristics with embedded interactions, a form of configural information processing (Hitt and Barr, 1989; Brown and Solomon, 1990, 1991; Leung and Trotman, 2005).

### Method

#### Participants

A total of 434 business students from a large public university participated. Participants were recruited using the college’s Student Research Program, which provides students the opportunity to earn course credit by participating in social science studies. Students participated asynchronously, accessing experimental materials *via* their personal computers and web browsers. The consent form identified the tasks to be completed and a request to set aside 70–80 min to complete the study in one sitting. Review of total time spent by each participant indicated a low of 6.15 min and a high of 51 h, indicating that participants with extreme times (less than 15 min and more than 2 h) either did not exert reasonable effort to complete the study or failed to follow instructions regarding completing in one sitting. Thus, we eliminate those participants (*n* = 20) from our data analysis, reducing the sample size to 414. Sixty percent of the participants were upper-division students (juniors and seniors); the remaining were lower-division students (33 %) and first-year master students (7 %). Fifty percent identified as female, the remaining identified as male.

#### Procedures

Participants began the experimental session by completing the cue processing task (described below), then an 11-item cognitive reflection test (CRT), the new Matching Familiar Figures Test (MFFT-2021), and a composite 10-item heuristics-and-biases task. The final section of the experiment included questions measuring thinking dispositions that Viator et al. (2020) reported correlating with MFFT performance: actively open-minded thinking and need for cognition. The study concluded with demographics questions. Below, we provide additional information on experimental materials.

#### Materials and measures

##### Matching Familiar Figures Test

Participants first completed two practice trials and then 20 test trials. Participants viewed figure sets one at a time and selected potential matches by clicking on figures. Participants could spend as much time, or as little time, as they chose. If participants made an incorrect selection, they attempted another possible match until they found the target for that set of figures (up to a maximum of six selections). Once participants identified the matching figure in a set, they proceeded to the next set. As done in prior studies employing the MFFT, we measured performance based on accuracy (fewer errors indicate more reflection/less impulsivity) and response time to first selection (slower response indicates more reflection/less impulsivity; Leshem and Glicksohn, 2007; Toplak et al., 2007; Perales et al., 2009; Huddy et al., 2017).

##### MFFT-2021 accuracy

Given that the maximum number of errors was 100 (20 sets of figures times five potential incorrect selections), we computed an MFFT-2021 Accuracy score as 100 (a perfect score) minus the total number of errors incurred.^7^ 13.53 percentage of participants obtained an accuracy score of 100, incurring no selection error across all 20 trials. The mean accuracy score was 86.95 (*SD* 11.62).

##### MFFT-2021 response time

As is customary for MFFT studies, we computed the time to the first response, a potential indicator of impulsivity; the mean Response Time per trial was 29.40 s (*SD* 18.79).

##### Cognitive reflection test (CRT-11) accuracy

Similar to prior studies, we use CRT Accuracy as a control variable. The CRT reportedly measures analytic vs. intuitive thinking (Pennycook et al., 2012, 2014a,b; Shenhav et al., 2012; Thompson and Johnson, 2014; Trippas et al., 2015; Weinhardt et al., 2015) and is associated with the avoidance of rational thinking errors (Viator et al., 2020). We used an 11-item CRT that included seven items developed by Toplak et al. (2014) and an additional four items developed by Thomson and Oppenheimer (2016), which rely less on numeracy skill. The mean CRT score was 4.76 (SD 2.36); the median was 4.0.

##### Heuristics-and-biases composite

For assessing avoidance of rational thinking errors, we used the 10 classic heuristic-and-biases tasks described in Viator et al. (2020).^8^ We scored normatively correct responses as one and zero otherwise. Higher summed scores indicate fewer rational thinking errors, with a potential maximum score of 10. The mean score was 5.19 (*SD* 2.06).

##### Cue processing task

This task required participants to evaluate 32 potential employers based on “guidelines specified by a close friend,” which included specific interactions of job characteristics. Configural information processing occurs when participants incorporate the specified interactions into their evaluation of potential employers (Hitt and Barr, 1989; Brown and Solomon, 1990, 1991; Leung and Trotman, 2005). Using recruiting signals assimilated by Banks et al. (2019), we selected four potential employer job characteristics: Team-based Culture, Rapid Advancement, Coaching and Guidance, and Work-life Balance. We also included Starting Salary as a fifth cue. We set each cue at two levels (Above Average and Below Average), yielding 32 cases (2^5^) that participants evaluated.

The scenario stated: “A close friend is graduating and wants your assistance in evaluating potential employers” based on five job characteristics that she/he considers to be important. The full set of instructions is posted at Open Science Framework.^9^ The potential for configural information processing was manipulated by specifying two potential interactions. First, the friend was interested in companies with above average Rapid Advancement, but conditional on the availability of Coaching and Guidance. Second, the friend, of course, preferred an above average Starting Salary, but conditional on the company’s commitment to Work-life Balance. Participants evaluated potential employers using an 11-point scale, from “Not Attractive” to “Very Attractive.” Participants completed two training cases that set each cue at the same level (all above average, and then all below average). After rating the extreme cases, participants were told that based on the friend’s guidelines the potential employer with all cues above average would have the highest rating of 10 (Very Attractive); conversely, the potential employer with all cues below average would have the lowest rating of 0 (Not Attractive). Participants were randomly assigned a starting case and then evaluated the full set of 32 cases. For the first 16 cases, after submitting an evaluation, participants viewed a screenshot of “the friend’s preferences,” in order to emphasize the two potential interactions.

The cue processing task did not have a predetermined normative response. Thus, similar to other studies of configural information processing (Leung and Trotman, 2005), we measured performance quality by comparing participant responses to a “gold standard,” which we derived from a pilot study that utilized only the configural processing task and the new MFFT-2021 task. 76 students participated. For each participant, we performed a regression analysis and identify the weight assigned to each cue and the two interactions terms. Nine of these participants obtained statistically significant weighting of the two interaction terms. However, only one of these participants provided statistically significant weights for both the main effects and the interaction terms (*r*^2^ = 0.956). Another participant obtained the highest *r*^2^ (0.983) but avoided assigning main effect weights to the two cues whose importance was conditional (Rapid Advancement and Starting Salary). We ran analyses using the responses from each of these two participants as the “gold standard” and observed comparable results. The analyses reported in the Results section are based on responses from the first participant identified above. We calculated two measures of performance.

##### Judgment achievement

Judgment Achievement is a lens model parameter calculated by correlating each participant’s evaluations with the evaluations provided by the “gold standard” participant from the pilot study. These correlations ranged from −0.269 to 0.961, with a median of 0.864. The mean was 0.791 (*SD* 0.218).

##### Absolute difference in weights

Utilizing the weighting of job characteristic cues generated by regression analysis, we calculated the absolute difference between a participant’s weighting of a parameter and the weighting provided by the “gold standard” participant in the pilot study. For each participant, we summed the absolute differences across each parameter (intercept, main effects, and interaction terms) to obtain the Absolute Difference in Weights, which had a mean of 9.611 (*SD* 2.812).

##### Actively open-minded thinking

We used an eight-item Actively open-minded thinking (AOT) scale, which is self-reported and measures individuals’ beliefs regarding issues such as “people should revise their beliefs in response to new information or evidence” and “changing your mind is a sign of weakness” (reverse scored; Haran et al., 2013; Baron et al., 2015). The mean score was 33.42 (*SD* 5.22).

##### Need for cognition

We used an 18-item Need for cognition (NFC) scale, which is a self-reported measure of the disposition to think abstractly, attempt challenging problems, and generate new solutions (Cacioppo et al., 1996; Wedell, 2011). The mean score was 72.29 (*SD* 11.69).

### Results

#### Convergent validity: Comparison of MFFT-2021 and MFFT20 performance characteristics

Table 1 presents a comparison of fundamental characteristics. Participants using the MFFT-2021, on average, spent more minutes on the task (*M* = 10.85, *SD* = 6.10) and more seconds selecting their first response (*M* = 29.40, *SD* = 18.79) compared to participants using the MFFT20 in the Viator et al. (2020) study (*M* = 8.43, *SD* = 3.12 and *M* = 21.22, *SD* = 9.87, respectively). However, the new figures appear to be more challenging, with average incorrect responses of 13.05 (*SD* = 11.62), compared to 9.26 (*SD* = 8.12) for the MFFT20. Nonetheless, 13.53% of participants completed the MFFT-2021 with 100% correct responses, compared to 9.89% for the MFFT20. As noted in Table 1, each *t* test was statistically significant (*p* < 0.0001).

**Table 1:** Comparison of MFFT response characteristics by version.

MFFT characteristics	MFFT-2021	MFFT20	Test statistic	Value of *p*
Number of participants	414	435		
Average total minutes spent on MFFT task	10.85	8.43	*t*(847) = 7.32	0.0001
Average number of seconds to first response	29.40	21.22	*t*(847) = 8.00	0.0001
Average number of incorrect responses	13.05	9.26	*t*(847) = 5.53	0.0001
Percentage of participants with 100% correct responses	13.53	9.89%	χ^2^ = 2.73	n.s.

Given apparent differences in SDs, we tested for equality of variances (Levene’s test) and found unequal variances across all three measures (*p* < 0.0001). Specifically, participants using the MFFT-2021 demonstrated more variance in total minutes spent on the task, average number of seconds to first response, and number of incorrect response. However, because of the large sample sizes, adjusting for unequal variances did not change the *p* values reported in Table 1, which was highly significant. Nonetheless, the driver of the increased variance remains unclear and is discussed as a limitation in the Discussion section below.

The coherence in these results provides partial evidence of convergent validity: we would expect that participants engaging in reflection, versus responding impulsively, would spend more time reviewing the figures prior to selecting their first response, and, in general, spend more time on the task. Further, the MFFT-2021 appears to reduce the ceiling effect, given that the average number of incorrect responses was higher compared to the MFFT20. Finally, we note that the chi-square test for percentage of participants with 100% correct responses yielded an insignificant *p* value of 0.098.

#### Correlation analysis

Table 2 presents zero-order correlations for the variables in the current study. Our primary focus is the correlations presented in the first column, which are correlations of MFFT-2021 Accuracy with the remaining variables. Each of these correlations is statistically significant (*p* < 0.0001). Most notable are correlations with the outcome variables for the current study: Heuristics-and-biases composite (*r* = 0.358), Judgment Achievement (*r* = 0.396), and Absolute Difference in Weights (*r* = −0.335), supporting predictive validity for the MFFT-2021. Furthermore, MFFT-2021 Accuracy is correlated with variables previously demonstrated to be antecedents to reflection (Viator et al., 2020): Actively Open-minded Thinking (*r* = 0.244) and Need for Cognition (*r* = 0.186). Finally, we note that MFFT-2021 Accuracy is correlated with CRT-11 Accuracy (*r* = 0.355), a measure of analytic thinking.

**Table 2:** Study 1 Means, SDs, and zero-order correlations.

		1.	2.	3.	4.	5.	6.	7.	8.
1.	MFFT-2021 Accuracy	—							
2.	MFFT-2021 Response Time	0.504^***^	—						
3.	CRT-11 Accuracy	0.355^***^	0.192^***^	—					
4.	Actively Open-minded Thinking	0.244^***^	0.121^*^	0.273^***^	—				
5.	Need for Cognition	0.186^***^	0.078	0.211^***^	0.313^***^	—			
6.	Heuristics-and-biases Composite	0.358^***^	0.242^***^	0.454^***^	0.360^***^	0.234^***^	—		
7.	Judgment Achievement^a^	0.396^***^	0.173^***^	0.267^***^	0.214^***^	0.047	0.307^***^	—	
8.	Absolute Difference in Weights^a^	−0.335^***^	−0.209^***^	−0.218^***^	−0.122^*^	0.024	−0.267^***^	−0.682^***^	—
	***M***	86.95	29.40	4.76	33.42	72.29	5.19	0.79	9.61
	**(*SD*)**	(11.62)	(18.79)	(2.36)	(5.22)	(11.69)	(2.06)	(0.218)	(2.81)

a Both Judgment Accuracy and Absolute Difference in Weights measure the quality of configural information processing compared to “gold standard” a pilot-study participant who provided statistically significant weights for both the main effects and the interaction terms.

*** <0.001;

** <0.01;

* <0.05.

*N* = 414.

Table 3 presents a comparison of key correlation coefficients that further examine the convergent validity for the MFFT-2021. The correlations for both MFFT Accuracy and Response Time with the previously mentioned antecedents to reflection (Actively Open-minded Thinking and Need for Cognition) tended to be higher for the MFFT-2021 compared to those for the MFFT20. This pattern supports convergent validity, given that higher levels of thinking dispositions (Actively Open-minded Thinking and Need for Cognition) should be associated with higher levels of reflection (less impulsivity). The notable exception to this pattern was the correlation of MFFT Response Time with Need for Cognition, which was lower for the MFFT-2021 vs. MFFT20. Although the movements toward stronger correlations would support convergent validity, we note that statistical tests using Fisher’s *r* to *z* transformation indicates that the differences in correlations (i.e., MFFT-2021 correlations compared to MFFT20 correlations) are not statistically significant (*α* = 0.05). Thus, we observe a trend in the expected direction, but the difference in magnitude was not statistically significant. We note a similar trend in the correlations with CRT-7 Accuracy.

**Table 3:** Comparison of MFFT correlations by version.

		MFFT accuracy		MFFT response time	
		MFFT-2021^a^	MFFT20^b^	MFFT-2021^a^	MFFT20^b^
1.	MFFT Accuracy	—	—		
2.	MFFT Response Time	0.504^***^	0.677^***^	—	—
3.	CRT-7^c^ Accuracy	0.325^***^	0.252^***^	0.168^***^	0.145^**^
4.	Actively Open-minded Thinking	0.244^***^	0.116^*^	0.121^*^	0.058
5.	Need for Cognition	0.186^***^	0.125^**^	0.078	0.107^*^

*** <0.001;

** <0.01;

* <0.05.

a *N* = 414.

b *N* = 435.

c The CRT-7, which is a subset of the CRT-11, was used in the current study and the Viator et al. (2020) study.

Table 3 also presents the correlation of MFFT Accuracy and MFFT Response Time for the two versions. The correlation was lower for the MFFT-2021 (*r* = 0.504) vs. the MFFT20 (*r* = 0.677). Based on statistical tests using Fisher’s *r* to *z* transformation, the difference between the two correlations is statistically significant (*p* < 0.0001) and provides further evidence that the MFFT-2021 is more challenging than the MFFT20: spending additional time reviewing the figures, in itself, was not sufficient for avoiding response errors.

#### Predictive validity: Regression analysis (Avoidance of rational thinking errors)

We perform regression analysis to test the predictive validity of the MFFT-2021. As previously noted, the heuristics-and-biases composite was employed as an outcome variable in both the current study and the Viator et al. (2020) study. Table 4 presents results of regressing the heuristics-and-biases composite on the two predictor variables used in both studies: MFFT Accuracy and CRT Accuracy. MFFT Accuracy was a statistically significant predictor in the current study (*B* = 0.0400, *SE* = 0.0081) and the prior study (*B* = 0.0311, *SE* = 0.0109), providing explanation of performance on the heuristics-and-biases task beyond that provided by CRT Accuracy. We note that replacing the CRT-11 with the CRT-7 (a subset of the CRT-11 that was used in the study by Viator et al., 2020) yielded results (untabulated) that are essentially identical to the results reported in Table 4. These analyses suggest that MFFT-2021 Accuracy remains a reliable predictor of the tendency to avoid rational thinking errors.

**Table 4:** Study 1 Regression analysis of heuristics-and-biases composite.

Studies and predictor variables	*B*	*SE*	*t*-statistic	Value of *p*
Current study (*N* = 414; *R*^2^ = 0.251)				
Intercept	0.1549	0.6677	0.23	0.8166
MFFT-2021 accuracy	0.0400	0.0081	4.92	0.0001
CRT-11 accuracy	0.3274	0.0399	8.20	0.0001
Viator et al. (2020) study (*N* = 435; *R*^2^ = 0.272)				
Intercept	1.4787	0.9663	1.53	0.1267
MFFT20 accuracy	0.0311	0.0109	2.87	0.0044
CRT-7 accuracy	0.5424	0.0482	11.26	0.0001

#### Predictive validity: Regression analysis (Configural information processing)

##### Judgment achievement

Table 5 presents results of regressing Judgment Achievement on the two predictor variables and their interaction. Both MFFT-2021 Accuracy and CRT-11 Accuracy were statistically significant (*B* = 0.0112, *SE* = 0.0018 and *B* = 0.1115, *SE* = 0.0327, respectively), indicating that *both* higher reflection (fewer errors on the MFFT-2021) *and* higher analytic thinking (higher correct responses on the CRT-11) were associated with higher levels of configural information processing (i.e., stronger correlations with the “gold standard” performer). The regression analysis yielded an interaction effect previously unobserved (*B* = −0.0011, *SE* = 0.0004), suggesting that the impact of reflection (versus impulsivity) and analytical thinking (versus intuitive thinking) on configural information processing is somewhat curvilinear, such that higher levels of both thinking styles have a diminishing marginal effect on configural information processing.

**Table 5:** Study 1 Regression analysis of configural information processing.

Outcome and predictor variables	*B*	*SE*	*t*-statistic	Value of *p*
Judgment Achievement (*N* = 414; *R*^2^ = 0.193)				
Intercept	−0.2432	0.1539	−1.58	0.1147
MFFT-2021 accuracy	0.0112	0.0018	6.22	0.0001
CRT-11 accuracy	0.1115	0.0327	3.41	0.0007
MFFT-2021 accuracy ^*^ CRT-11 accuracy	−0.0011	0.0004	−3.03	0.0026
Absolute Difference in Weights (*N* = 414; *R*^2^ = 0.123)				
Intercept	16.4590	0.9848	16.71	0.0001
MFFT-2021 accuracy	−0.0714	0.0120	−5.97	0.0001
CRT-11 accuracy	−0.1348	0.0589	−2.29	0.0225

*N* = 414.

##### Absolute difference in weights

In Table 5, we also present results of regressing Absolute Difference in Weights on the two predictor variables. We note that there was no interaction effect. Both MFFT-2021 Accuracy and CRT-11 Accuracy were statistically significant (*B* = −0.0714, *SE* = 0.0120 and *B* = −0.1348, *SE* = 0.0589, respectively); higher reflection and higher analytic thinking were associated with lower absolute differences between the cue weights provided by participants and the cue weights provided by the “gold standard” performer. These results provide additional evidence supporting the predictive validity of the MFFT-2021.

#### Concurrent validity test

In concurrent validity testing, we expect to observe levels of MFFT Accuracy and Response Time that differ across groups of participants in a predictable manner. We placed participants into three groups based on CRT performance, given that CRT Accuracy tends to be associated with reflection rather than impulsivity (Baron et al., 2015; Alos-Ferrer et al., 2016; Jelihovschi et al., 2018). Specifically, we expected that participants scoring higher (lower) on the CRT would have higher (lower) MFFT Accuracy scores and longer (shorter) MFFT Response Times. The three groups identified are: High CRT Performance (accuracy was greater than 5 out of 11; 35.3% of participants), Medium CRT Performance (accuracy was either 4 or 5 out of 11; 30.9% of participants), and Low CRT Performance (accuracy was less than 4 out of 11; 33.8% of participants). The ANOVAs for both MFFT Accuracy and Response Time were statistically significant [*F* (2, 411) = 21.71, *p* < 0.0001 and *F* (2, 411) = 5.78, *p* < 0.004, respectively]. Based on planned comparison tests (alpha = 0.05), MFFT Accuracy was statistically different across the three groups, such that participants with High CRT Performance also obtained relatively high MFFT scores (90.8), participants with Medium CRT Performance obtained medium MFFT scores (87.6), and participants with Low CRT Performance obtained low MFFT scores (82.3). Planned comparison tests for MFFT Response Time indicated that participants with High CRT Performance demonstrated relatively slow response times (33.4 s) that were statistically different (alpha = 0.05) from the other two groups (28.4 and 26.1 for Medium CRT Performance and Low CRT Performance, respectively); however, response time for the latter two groups was not statistically different. The results support the assertion that MFFT-2021 Accuracy scores, and to a less extent MFFT-2021 Response Time, differed in a predictable manner across participant groups, supporting concurrent validity.

### Discussion

In Study 1, we found evidence supporting convergent, predictive, and concurrent validity for the MFFT-2021. Participants responding to figures in the MFFT-2021 were more engaged (spent more time on the task), took more time in making their first response, and were more likely to complete the task without errors. We also note that the MFFT-2021 appears to be more challenging, given that the average number of errors was higher than in the comparison group. However, one limitation is that participants in the current study completed all tasks asynchronously, accessing experimental materials *via* their personal computers and web browsers, whereas participants in the Viator et al. (2020) study participated in a controlled environment (i.e., the college’s behavioral research lab, which was available prior to the Covid-19 pandemic). The opportunity to participate asynchronously and unsupervised might have increased the number of participants who have an impulsive cognitive style, thus increasing the average number of errors per participant in the current study. Furthermore, as previously noted, the variances for three key metrics (total time spent on the MFFT task, average number of seconds to first response, and average number of incorrect responses) were higher in the current study compared to Viator et al. (2020). This increase in variance might be attributed to differences in participation method (online versus behavioral lab) rather than differences in MFFT versions. However, even though differences in participation methods is a concern, we note that participants in the current study, on average, spent more total time on the MFFT-2021 task and exhibited greater latency in their first response. Further, we note that prior research comparing responses of online participants (recruited *via* MTurk and social media postings) to responses from face-to-face/lab participation has found comparable and indistinguishable results (Casler et al., 2013).

Most importantly, the MFFT-2021 remained a reliable predictor of rational thinking, such that participants who demonstrated more reflection (less impulsivity) on the MFFT-2021 tended to avoid rational thinking errors, as measured by the 10-item heuristics-and-biases composite. Furthermore, performance on the MFFT-2021 predicted higher quality judgments in assessing job characteristic cues with embedded interactions, a form of configural information processing. Finally, we found evidence of concurrent validity; performance on the MFFT-2021 differed in a predictable manner for participants grouped by CRT performance.

## Study 2

**Figure 2:**
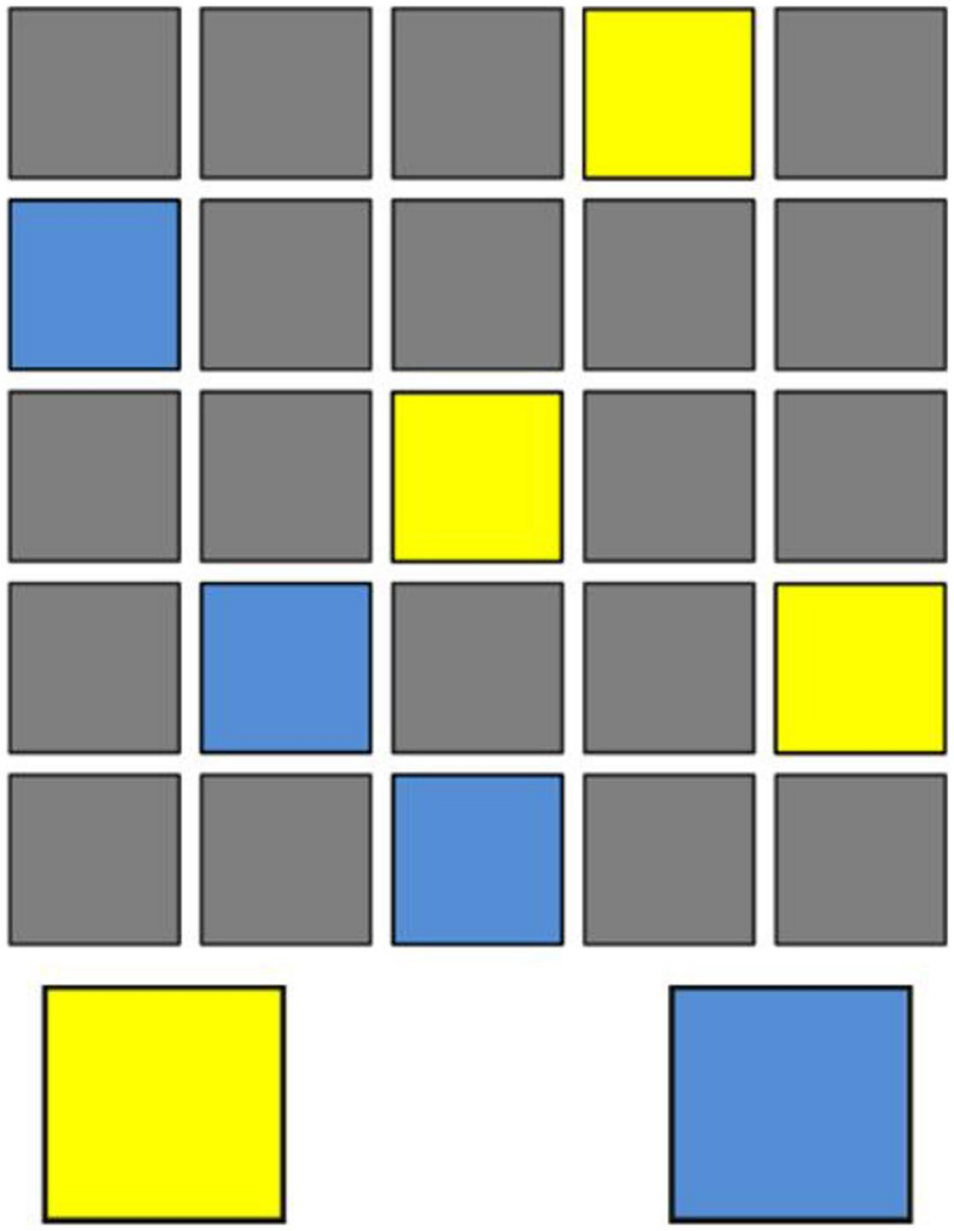
Example matrix from Information Sampling Task.

One criticism of the MFFT as a measure of reflection-impulsivity is that the task places a relatively high demand on visual search and visual working memory (Clark et al., 2009). An alternative behavioral measure of reflection-impulsivity is the Information Sampling Task (IST). In the IST, participants view a 5 × 5 matrix that conceals boxes having one of two colors, such as yellow or blue (See Figure 2 for an example IST matrix.) Participants open boxes one at a time and ultimately choose which of the two colors is in the majority. Working memory load is limited, given that boxes remain open until a decision is rendered. Using two different versions of the instrument (Fixed Win and Reward Conflict, described below), prior studies have found a positive correlation between the number of boxes opened (the amount of information sampled) and correct judgments. Clark et al. (2009) argue that this pattern is evidence of reflection-impulsivity: at the extremes, impulsive participants tend to open fewer boxes and obtain fewer correct decisions, while reflective participants tend to open more boxes and obtain more correct decisions.

Critical to our study, Clark et al. (2003) reported a statistically significant association between slow, accurate responses on the MFFT and performance on the IST (number of boxes opened and probability of being correct), indicating concurrent validity, such that both tasks appear to measure reflection-impulsivity. Furthermore, in an extensive study of different behavioral and self-reported measures of impulsivity, Caswell et al. (2015) identified that measures from both the MFFT and IST loaded on a single factor [identified as reflection-impulsivity (RI)], which was separate from other measures of impulsivity. The authors note that although the MFFT has been criticized for confounding behavioral impulsivity with other cognitive processes, thus inspiring development of the IST, the results of their study suggest that the MFFT and the IST “index the same primary underlying process” (p. 72). We examine through exploratory factor analysis whether the MFFT-2021 and the IST continue to load on a single factor, or not, which is a test of discriminate validity, such that loading on separate factors would indicate that these two behavioral measures identify different subtypes of reflection-impulsivity. Further, these two behavioral measures may differ in their relative strength in predicting specific outcomes, such as the avoidance of rational thinking errors. We are not aware of any prior study that has examined the relative predictive strength of the MFFT versus the IST; thus, testing whether the MFFT-2021 and the IST are comparable in predicting the avoidance of rational thinking errors is unique. Furthermore, we examine whether these two behavioral measures of reflection-impulsivity are stronger predictors compared to a less time-consuming self-reported measure of impulsivity, the Barratt Impulsiveness Scale (BIS-11; Patton et al., 1995).^10^

### Method

#### Participants

We recruited an additional 193 business students, using the methods reported in Study 1. Five participants provided incomplete data and three participants did not follow the instructions for completing the study in one sitting, yielding 185 usable responses. 77.8 percent of the participants were upper-division students (juniors and seniors); the remaining were lower-division students (22.2 percent). 43.2 percent (55.1 percent) identified as female (male) and 1.7 percent identified as either non-binary or declined to answer.

#### Procedures

We randomized presentation of the MFFT-2021 and the IST. 53.5 percent of the participants completed the MFFT-2021 first, prior to completing the CRT and then the IST. The other 46.5 percent completed the IST first. All participants then completed the 10-item heuristics-and-biases composite task. In final phase of the experiment, we randomized presentation of the BIS-11 and measures of thinking dispositions (need for cognition and actively open-minded thinking); 50.8 percent (49.2 percent) completed the BIS-11 first (second). Participants then completed demographics questions. Below, we provide additional information regarding the IST and the BIS-11, which were not utilized in Study 1.

#### Materials and measures

##### Information sampling task

As previously noted, participants view a 5 × 5 matrix that conceals boxes having one of two colors. Participants open boxes one at a time and ultimately choose which of the two colors is in the majority. Clark and colleagues provide detail instructions regarding the construction and operation of the IST (Clark et al., 2006, 2009) and Caswell et al. (2013b) provide example screen displays (p. 327). We developed and implemented a version of the IST using software tools available at www.PsyToolkit.org (Stoet, 2010, 2017). Similar to our implementation of the MFFT-2021, the images utilized and the computer code are available at Open Science Framework.^11^ A demonstration version is available at https://us.psytoolkit.org/c/3.4.0/survey?s=HwPEx.

The IST utilizes two versions of the task with 10 trials each. In the Fixed Win version (FW), participants win 100 points for correct decisions regardless of the number of boxed opened; they lose 100 points for an incorrect decision. In the Reward Conflict version (RC), participants start each trial with 250 points and lose 10 points for each box opened. Participants win the remaining points with a correct decision; otherwise, they lose 100 points for an incorrect decision. For each participant, we recorded and report average probability of being correct at point of decision [*P* (correct)], Accuracy (the number of correct decisions out of 20 decisions), Average Number of Boxes Opened, and Average Latency of box opening (number of boxes opened divided by time to make a decision; Clark et al., 2006).

##### Barratt impulsiveness scale, version 11

The BIS-11 is a 30-item self-reported measure of impulsivity (Patton et al., 1995). The scale has 10 items for each of three subscores for impulsivity: attentional (e.g., “I do not pay attention”), motor (e.g., “I do things without thinking”), and non-planning (e.g., “I am more interested in the present than the future”). Participants respond using a four-point scale, such that higher summed scores indicates higher levels of impulsiveness.

### Results

#### Correlation analysis

Table 6 presents zero-order correlations for the variables used in Study 2. Our primary focus is the correlations presented in the bottom row, which are correlations of performance on the heuristics-and-biases task with the other variables. We note that the two MFFT-2021 measures (Accuracy and Response Time) and the four IST measures [*P (correct),* Accuracy, Average Number of Boxes Opened, and Average Latency] have statistically significant positive correlations with performance on the heuristics-and-biases task, indicating strong predictive validity. Two BIS-11 subtypes exhibited either no correlation or modest correlation (Attention and Motor subtypes, respectively); only the Non-planning subtype had a negative correlation with a *p* value less than 0.05. The correlations between the heuristics-and-biases task and the remaining variables (CRT-11 Accuracy, actively open-minded thinking, and need for cognition) were positive and comparable to those reported in Study 1.

**Table 6:** Study 2 Means, SDs, and zero-order correlations.

		1.	2.	3.	4.	5.	6.	7.	8.	9.	10.	11.	12.	13.
1.	MFFT-2021 Accuracy	—												
2.	MFFT-2021 Response Time	0.724^***^	—											
3.	IST Average *P*(correct)	0.407^***^	0.293^***^	—										
4.	IST Accuracy	0.351^***^	0.270^***^	0.802^***^	—									
5.	IST Average Number of Boxes Opened	0.325^***^	0.281^***^	0.944^***^	0.765^***^	—								
6.	IST Average Latency	0.157^*^	0.009	0.627^***^	0.553^***^	0.631^***^	—							
7.	BIS Attention	−0.033	0.077	−0.121	−0.137	−0.096	−0.071	—						
8.	BIS Motor	−0.164^*^	−0.094	−0.187^**^	−0.240^***^	−0.143	−0.132	0.634^***^	—					
9.	BIS Non-planning	−0.211^**^	−0.102	−0.177^*^	−0.157^*^	−0.159^*^	−0.109	0.456^***^	0.613^***^	—				
10.	CRT-11 Accuracy	0.292^***^	0.217^**^	0.272^***^	0.184^*^	0.219^**^	0.267^***^	0.042	−0.152^*^	−0.122	—			
11.	Actively Open-minded Thinking	0.346^***^	0.246^***^	0.294^***^	0.218^**^	0.235^**^	0.245^***^	0.032	−0.168^*^	−0.180^*^	0.387^***^	—		
12.	Need for Cognition	0.247^***^	0.216^**^	0.170^*^	0.186^*^	0.185^*^	0.155^*^	−0.118	−0.331^***^	−0.379^***^	0.217^**^	0.248^***^	—	
13.	Heuristics-and-biases Composite	0.342^***^	0.273^***^	0.301^***^	0.251^***^	0.236^**^	0.250^***^	−0.008	−0.128	−0.167^*^	0.532^***^	0.444^***^	0.180^*^	—
	***M***	85.52	20.73	0.73	14.66	10.51	1.08	22.76	21.04	20.97	5.08	33.20	71.85	5.45
	**(*SD*)**	(12.88)	(12.06)	(0.12)	(3.05)	(5.88)	(0.54)	(3.98)	(4.17)	(4.21)	(2.39)	(5.44)	(12.43)	(2.12)

*** <0.001;

** <0.01;

* <0.05.

*N* = 185.

#### Discriminant validity: Factor analysis

We conducted an exploratory factor analysis using Geomin oblique rotation, which allows factors to be correlated. A three-factor model was indicated and appeared to fit the data reasonably well. The Comparative Fit Index (CFI) and the Tucker Lewis Index (TLI) were 0.974 and 0.936, respectively; however, the root mean square error of approximation (RMSEA) was 0.093 with a 90% confidence interval of 0.060–0.126 and thus unlikely to be less than the target of 0.05. Table 7 presents the identified factor structures. All four IST measures loaded on Factor 1 with relatively high correlations, ranging from 0.644 to 0.987; both MFFT measures loaded on Factor 2 with relatively high correlations of 0.993 and 0.730. The finding of these two separate factors, one based on the MFFT and the other based on the IST, supports discriminant validity and suggests that the two measures indicate different subtypes of reflection-impulsivity. Factor 3 represents self-reported impulsivity; each of the three BIS-11 subtypes had relatively high correlations, ranging from 0.659 to 0.932. We note that the Geomin rotated factor loadings indicated that CRT Accuracy had statistically significant (*p* < 0.05) loadings on both Factor 1 and Factor 2; however, as shown in Table 7, the correlation of CRT Accuracy with each factor is very low (0.265 and 0.293), suggesting that the CRT is not a behavioral measure of reflection-impulsivity.^12^ The inter-factor correlations were modest, ranging from-0.222 to 0.397.

**Table 7:** Study 2 Factor loadings after Geomin oblique rotation.

Variables	Factor 1	Factor 2	Factor 3
MFFT-2021 Accuracy	0.392	**0.993**	−0.183
MFFT-2021 Response Time	0.292	**0.730**	−0.086
IST Average *P*(correct)	**0.987**	0.412	−0.216
IST Accuracy	**0.811**	0.354	−0.259
IST Average Number of Boxes Opened	**0.958**	0.330	−0.171
IST Average Latency	**0.644**	0.157	−0.150
BIS Attention	−0.124	−0.024	**0.681**
BIS Motor	−0.192	−0.155	**0.932**
BIS Non-planning	−0.182	−0.205	**0.659**
CRT-11 Accuracy	0.265	0.293	−0.145
Eigenvalues	3.839	1.956	1.501

*N* = 185.

Bold values indicate relatively high factor loadings.

#### Predictive validity: Regression analysis (avoidance of rational thinking errors)

We used stepwise regression to examine which variables are relatively stronger predictors of performance on the heuristics-and-biases task. The analysis included the 10 potential predictor variables listed in Table 6: two MFFT-2021 measures, four IST measures, three BIS-11 subtypes, and CRT Accuracy. We used the SAS default cutoffs for adding variables to the model (*p* = 0.05) and for removing variables from the model (*p* = 0.05). The process generated Model 1 shown in Table 8, in which MFFT-2021 Accuracy is a statistically significant predictor (*B* = 0.0337, *SE* = 0.0105), in addition to CRT Accuracy (*B* = 0.4190, *SE* = 0.0567).

**Table 8:** Study 2 Regression analysis of heuristics-and-biases composite.

Models and predictor variables	*B*	*SE*	*t*-statistic	Value of *p*
Model 1 (*R*^2^ = 0.321, *F* = 42.99)				
Intercept	0.4463	0.8713	0.51	0.6091
MFFT-2021 accuracy	0.0337	0.0105	3.20	0.0016
CRT-11 accuracy	0.4190	0.0567	7.39	0.0001
Model 2 (*R*^2^ = 0.331; *F* = 29.82)				
Intercept	−0.3041	0.9805	−0.31	0.7568
MFFT-2021 accuracy	0.0271	0.0112	2.42	0.0166
CRT-11 accuracy	0.4025	0.0573	7.02	0.0001
IST Average *P*(correct)	1.912	1.1656	1.64	0.1027
Model 3 (*R*^2^ = 0.331; *F* = 29.82)				
Intercept	−0.1070	0.932	−0.11	0.9087
MFFT-2021 accuracy	0.0280	0.0111	2.53	0.0122
CRT-11 accuracy	0.4106	0.0567	7.25	0.0001
IST Accuracy	0.0738	0.0454	1.62	0.1063

*N* = 185.

We directly examined whether adding any of the remaining variables one at a time (the four IST measures and the three BIS-11 measures) further improved model fit. No statistically significant improvement was detected. However, Model 2 and Model 3 in Table 8 show that both IST *P* (correct) and IST Accuracy approach providing additional explanation of variation in the avoidance of rational thinking errors and are close to being statistically significant (*p* = 0.1027 and *p* = 0.1063, respectively). Thus, we note that IST measures should not be dismissed as predictors of rational thinking (avoidance of rational thinking errors). We ran four separate models, each with one IST measure and the control variable CRT Accuracy. The untabulated results indicated that two IST measures provided additional information beyond that provided by the CRT: both IST *P* (correct) and IST Accuracy were statistically significant (*p* values less than 0.01 and 0.02, respectively), after controlling for CRT Accuracy.

### Discussion

Study 2 provided evidence supporting the reliability of the MFFT-2021; MFFT Accuracy remained a reliable predictor of performance on the heuristics-and-biases composite task. Furthermore, compared to IST measures of reflection-impulsivity, MFFT Accuracy was a stronger predictor of performance. Contrary to the findings of Caswell et al. (2015), our exploratory factor analysis yielded two separate factors for (the MFFT-2021 and the IST), supporting discriminant validity and suggesting that these two instruments measure different subtypes of reflection-impulsivity.

## General discussion

Several limitations are worth noting. Our participant pools consisted primarily of undergraduate college of business students, whose responses and performance might differ from the general population, even when controlling for age. However, our participant pool is consistent with those of Viator et al. (2020), our primary comparison group. Our primary outcome variable (avoidance of rational thinking errors) is quite different from other studies that measure the relationship between reflection-impulsivity and behaviors such as anger control in adults with ADHD (McDonagh et al., 2019), physical aggression (Sanchez-Martin et al., 2011), use of illegal substances (Huddy et al., 2017), computerized adaptive testing (Wang and Lu, 2018), risk-taking behavior (Young et al., 2013), and, most importantly, studies of psychopharmacology and addictive behaviors, which have extensively used the IST for measuring reflection-impulsivity (Clark et al., 2009; Caswell et al., 2016; Harris et al., 2018; Frydman et al., 2020). However, we did find evidence that reflection-impulsivity as measured by the MFFT-2021 remained a reliable predictor of performance, such that participants who demonstrated more reflection (less impulsivity) on the MFFT-2021 tended to avoid rational thinking errors, as measured by the 10-item heuristics-and-biases composite, and provided higher quality judgments in assessing job characteristic cues with embedded interactions, a form of configural information processing.

We conclude by noting that the MFFT-2021 meets three design criteria: the images are oriented for adults and adolescents, the images reflect the culture of the 21st century, and the images reside in an easily accessible repository. Our initial testing indicates that participants interacting with the MFFT-2021 were more engaged (spent more time on the task), took more time in making their first response, and were more likely to complete the task without errors, even though the average number of errors was higher than in the comparison group interacting with the original MFFT images. Although our objective in revising the MFFT was to generate figures that are familiar to adults and adolescents, the possibility remains that these figures might be suitable for studies of reflection-impulsivity in children, such as recent studies of music programs for pre-school children (Bugos and DeMarie, 2017), epilepsy in children (Lima et al., 2017), and sleep issues for children with ADHD (Lee et al., 2014).

## Data availability statement

The raw data supporting the conclusions of this article will be made available by the authors, without undue reservation.

## Ethics statement

The studies involving human participants were reviewed and approved by Human Research Protection Program. The participants provided their written informed consent to participate in this study.

## Author contributions

AV created the MFFT-2021 figures. RV and YJW designed and conducted the experiments. RV performed the statistical analyses and wrote the first drafts. YJW provided several critical revisions of the manuscript. All authors contributed to the article and approved the submitted version.

## Conflict of interest

AV was employed by company Dallas Art Therapy.

The remaining authors declare that the research was conducted in the absence of any commercial or financial relationships that could be construed as a potential conflict of interest.

## Publisher’s note

All claims expressed in this article are solely those of the authors and do not necessarily represent those of their affiliated organizations, or those of the publisher, the editors and the reviewers. Any product that may be evaluated in this article, or claim that may be made by its manufacturer, is not guaranteed or endorsed by the publisher.

## Footnotes

^1^For example, consider the distribution of error rates on a relatively easy MFFT. If the upper end of the distribution is relatively low, then a ceiling effect occurs, masking variation in participants’ tendency for impulsivity.

^2^Specifically, in developing the MFFT-2021, we avoided using images that are cartoonish, exaggerated, and clearly oriented for children, such as an imaginary animal, a walking leaf, a child-friendly toy duck, and the well-known child’s teddy bear in a chair. Also, we dropped images that would appear odd in the 21^st^ century, such as a landline telephone, dresses from the early 20^th^ century, and Native American tepees, which is probably culturally inappropriate for the 21^st^ century.

^3^Once a primary image was selected, non-matching figures were derived using 10 fundamental rules: (1) inserting new lines, removing lines, or changing the curvature of lines (2) adding or removing ornaments or other features, such as an animal’s tail (3) changing the angle of a feature, such as leaves on a plant (4) changing the position of an ornament, such as moving from the front of an object to the back (5) inverting an ornament or feature (6) changing the shape of an ornament, such as a circle becoming a rectangle (7) changing the proportional size of an ornament, such as petals on a flower (8) reversing an image, such as flipping the cuts on a key from right-side to left-side (9) adding or removing shading, and (10) changing a number, such as a 9 becoming a 6, or changing the order of numbers, such as 32 becoming 23.

^4^ http://acctweb.ba.ttu.edu/demo/default2.aspx

^5^The third co-author developed the new target figures by reviewing images from the original MFFT20 and selecting images from https://pixabay.com (a media website for sharing copyright free images). The selected images retained key features from prior versions of the MFFT. Detailed documentation of this selection process is available at Open Science Framework (https://osf.io/89gc6).

^6^ https://osf.io/7v4yg

^7^Reporting an accuracy score rather than an error score facilitates comparison with CRT accuracy and other measures reported in Study 2 (below).

^8^Viator et al. (2020) used two other measures of rational thinking: avoiding belief bias in syllogistic reasoning and avoiding ratio bias, which were less strongly correlated with performance on the MFFT.

^9^ https://osf.io/eznbt

^10^Prior studies have utilized the IST to measure reflection-impulsivity in the context of psychopharmacology issues (Crockett et al., 2012; Jepsen et al., 2018; Yang et al., 2018a,b; Herman et al., 2019), and addictive behaviors, including alcohol and substance abuse (Clark et al., 2006; Joos et al., 2013; Caswell et al., 2013a, 2016), obsessive–compulsive disorder (Frydman et al., 2020), and gambling (Harris et al., 2018).

^11^ https://osf.io/q47dm

^12^Prior research documents that the CRT is most likely a measure of intuitive versus analytic thinking (Pennycook et al., 2012, 2014a,b; Shenhav et al., 2012; Thompson and Johnson, 2014; Trippas et al., 2015; Weinhardt et al., 2015).
